# Improving fold resistance prediction of HIV-1 against protease and reverse transcriptase inhibitors using artificial neural networks

**DOI:** 10.1186/s12859-017-1782-x

**Published:** 2017-08-15

**Authors:** Olivier Sheik Amamuddy, Nigel T. Bishop, Özlem Tastan Bishop

**Affiliations:** 1 Research Unit in Bioinformatics (RUBi), Department of Biochemistry and Microbiology, Rhodes University, Grahamstown, 6140 South Africa; 2 Department of Mathematics (Pure and Applied), Rhodes University, Grahamstown, 6140 South Africa

**Keywords:** Artificial neural network, Drug resistance prediction, Subtype-specific training, HIV-1 subtype B, HIV reverse transcriptase, HIV protease

## Abstract

**Background:**

Drug resistance in HIV treatment is still a worldwide problem. Predicting resistance to antiretrovirals (ARVs) before starting any treatment is important. Prediction accuracy is essential, as low-accuracy predictions increase the risk of prescribing sub-optimal drug regimens leading to patients developing resistance sooner. Artificial Neural Networks (ANNs) are a powerful tool that would be able to assist in drug resistance prediction. In this study, we constrained the dataset to subtype B, sacrificing generalizability for a higher predictive performance, and demonstrated that the predictive quality of the ANN regression models have definite improvement for most ARVs.

**Results:**

Trained regression ANNs were optimized for eight protease inhibitors, six nucleoside reverse transcriptase (RT) inhibitors and four non-nucleoside RT inhibitors by experimenting combinations of rare variant filtering (none versus 1 residue occurrence) and ANN topologies (1–3 hidden layers with 2, 4, 6, 8 and 10 nodes per layer). Single hidden layers (5–20 nodes) were used for training where overfitting was detected. 5-fold cross-validation produced mean R^2^ values over 0.95 and standard deviations lower than 0.04 for all but two antiretrovirals.

**Conclusions:**

Overall, higher accuracies and lower variances (compared to results published in 2016) were obtained by experimenting with various preprocessing methods, while focusing on the most prevalent subtype in the raw dataset (subtype B).We thus highlight the need to develop and make available subtype-specific datasets for developing higher accuracy in drug-resistance prediction methods.

**Electronic supplementary material:**

The online version of this article (doi:10.1186/s12859-017-1782-x) contains supplementary material, which is available to authorized users.

## Background

Living with HIV has come a long way from being a deadly disease to become a manageable chronic infection [1] mainly due to the development and use of antiretrovirals (ARVs). However, resistance to ARVs still prevails for multiple reasons including non-adherence to treatment, use of sub-optimal regimens and delayed initiation of therapy [2, 3]. Thus predicting resistance to ARVs before and during any treatment is important, and therefore genotypic testing for prediction finds wide application due to its simplicity, speed and relatively low cost, in comparison to the gold standard of phenotypic assays [4–6]. Furthermore, the prediction algorithms are continuously evaluated [7, 8], while mutation lists keep being updated to improve predictability of drug resistance [9, 10]. Disparities between prediction methods have decreased but discordances still exist between the different algorithms, especially for some ARVs, as at 2015 [11]; which motivates the need to further improve accuracy.

Prediction accuracy is essential, as low-accuracy predictions increase the risk of prescribing sub-optimal drug regimens and missing the timing for regimen switches, leading to patients developing resistance sooner and so needing recourse to less well-tolerated third line ARV therapy. If left uncontrolled, the accumulation of resistance mutations may increase the probability of resistant strains directly spreading to drug-naive individuals, rendering therapy more difficult. In order to address these issues, different research groups have been involved in producing independent prediction algorithms – such as REGA [12], ANRS [13] and HIVdb [14] amongst others [15]. As stated in [17], to date the most widely used ones are the HIVdb algorithm [14] and the support vector machine-based geno2pheno tool [16, 18]. More recent work has applied different machine learning approaches for drug resistance prediction, for instance multi-label classification [17], K-Nearest Neighbor and Random Forests [19], sparse signal representations coupled to Delaunay triangulation [20, 21] and Support Vector Machines variants [22], some of which are based on sequence information, while others also utilise protein structural information.

The objective of this work was to develop prediction models that are as accurate as possible. This problem is usually treated as one of classification, since in a clinical context it is normally sufficient to predict the effectiveness (or not) of a given ARV. However, here we solve a regression problem, thereby making full use of all available data and so potentially improving the predictive accuracy of the model. We note that the model output may be transformed into a classification by setting cut-off values, and that the drug resistance score may be clinically useful if the value is borderline, i.e. very close to a cut-off value.

Our method incorporated the following features: (a) The prediction algorithm used was a regression Artificial Neural Network (ANN); (b) because the great majority of publicly available data in the Stanford HIVdb is for subtype B HIV, only subtype B data was used in this database to train and test the network, so that the prediction algorithm is mainly applicable to subtype B sequence data; (c) in order to reduce data noise, various forms of data filtering, as described in the Methodology section, were used. Our regression ANN models compared favourably against recent work by Shen and co-workers [19], for which similar metrics were used. The ANN regression models were applied to the protease (PR) inhibitors fosamprenavir (FPV), atazanavir (ATV), indinavir (IDV), lopinavir (LPV), saquinavir (SQV), tipranavir (TPV), nelfinavir (NFV) and darunavir (DRV), and to the reverse transcriptase (RT) inhibitors lamivudine (3TC), abacavir (ABC), zidovudine (AZT), stavudine (D4T), didanosine (DDI), tenofovir (TDF), efavirenz (EFV), etravirine (ETR), nevirapine (NVP), rilpivirine (RPV). Applying cut-offs, we obtain a classification output from our ANN models which is then evaluated against HIVdb and SHIVA [17]. Our work resulted in the production of drug-specific regression ANNs with high mean R^2^ values, low variance and competitive classification performances for each of the eight PR inhibitors (PIs), six nucleoside RT inhibitors (NRTIs) and four non-nucleoside RT inhibitors (NNRTIs) for predictions from subtype B HIV.

## Methods

### Dataset description

Unfiltered PhenoSense assay datasets were retrieved from Stanford HIVdb [23] for both PR and RT. The datasets are compactly organized from a consensus B sequence with conserved positions coded as “-”, with differing residues coded as the actual amino acids. Mixed residues are grouped together while indels are represented as “#” and “~” respectively in a tab-separated file format. Drug resistance scores for PR and RT inhibitors are present for each sequence entry as metadata.

### Dataset pre-processing

Incomplete sequence entries (i.e. with missing fold resistance ratios for some ARVs) were retained to increase the sample size. Sequences containing the ambiguous residue ‘X’, indels or the characters ‘.’, ‘*’, ‘l’, ‘d’ and ‘^’ were flagged and then expanded to obtain all possible sequences consistent with the sequence data. The sequence expansion procedure thus yielded differing numbers of sequences for each ARV (Table 1). Non-B subtypes were also filtered out from the dataset to improve predictability for the subtype B cluster only. RT sequences were truncated to 240 residues to conform to the format of the filtered RT PhenoSense dataset as available from Stanford HIVdb. Several sequence entries yielded several thousand to millions of combinations of sequences, which made the initial design non-practical in terms of running time and also potentially introduced bias to the model that would be obtained from the dataset. This inherent uncertainty resides in the fact that the sequences may truly be mixed or contain sequencing errors. Thus a filter was introduced that removed from the datasets any sequence whose expansion yielded more sequences than some user-chosen cut-off value.

**Table 1:** ANN topologies and filtering parameters for highest observed accuracies for the various ARVs

ARVs		Topology	Number of unique sequence IDs/expanded sequences	Number of allowed combinations	Rare variant filtering	Number of outliers removed
PIs	ATV	10x8x6	995 / 13,625	< 1000	✓	1
	DRV	8 × 8	590 / 10,374	< 1000	✓	2
	FPV	8x8x8	1429 / 17,501	< 1000	x	none
	IDV	8x6x10	1459 / 16,977	< 1000	✓	1
	LPV	10x8x10	1284 / 11,019	< 300	x	none
	NFV	10x10x10	1524 / 11,929	< 300	x	none
	SQV	10x10x8	1484 / 11,509	< 300	x	none
	TPV	10x6x8	698 / 11,989	< 1000	✓	2
NRTIs	3TC	10x10x6	1342 / 33,181	< 1000	✓	none
	ABC	14	1401 / 34,016	< 1000	x	none
	AZT	19	1358 / 33,818	< 1000	✓	none
	D4T	10x4x4	1365 / 34,056	< 1000	✓	none
	DDI	10x6x6	1368 / 34,062	< 1000	✓	none
	TDF	10 × 2	1130 / 29,637	< 1000	x	none
NNRTIs	EFV	10x6x10	1400 / 33,906	< 1000	✓	none
	ETR	8x2x10	448 / 11,397	< 1000	x	2
	NVP	10x10x4	1414 / 20,348	< 300	x	none
	RPV	16	169 / 2977	< 1000	✓	none

The experiment was initially started by training machine learners with sequences that had less than 5, 10, 20, 50, 100, 200, 300 and 1000 combinations upon expansion. Thereafter only the 300 and 1000 filter levels were used as candidates for rare variant filtering, due to their higher performance and number of unique sequence IDs that they contained. Rare variant filtering here means that a sequence is removed if it contains a residue at a given position that occurs only once across all sequence samples, and ANNs were constructed and tested both with and without this filtering. In order to process the sequence data, the amino acid letters were converted to integers using an ad hoc Python script, utilizing a simple integer encoding scheme, whereby residues “A”, “R”, “N”, “D”, “B”, “C”, “E”, “Q”, “Z”, “G”, “H”, “I”, “L”, “K”, “M”, “F”, “P”, “S”, “T”, “W”, “Y” and “V” were converted to positive integers 1 to 22 respectively in a similar manner, but not identical to the encoding approach used by Araya and Hazelhurst [4], who applied codon-based integer encoding instead on a dataset used by Ravela and coworkers in 2003 [24]. Possible outliers were detected by using (1) Principal Components Analysis from input features and target values and (2) the prediction error distributions between actual and predicted scores, and removed (Table 1).

### Neural network construction and architecture optimization

MATLAB’s (version 2016a) implementation of the Levenberg-Marquardt feed-forward algorithm with back-propagation from the Neural Network Toolbox was used for supervised training, utilizing the mean squared error (MSE) for weight adjustment. Absolutely conserved residue positions were filtered out in order to reduce computation time. The initial dataset was (pseudo) randomly split into training, testing and validation sets at rates of 70%, 15 and 15% respectively, setting random seed numbers for reproducibility in training and cross-validation. Training was stopped upon reaching any of a maximum of 1000 epochs, a maximum of 6 successive validation failures to decrease or a performance gradient lower than a minimum set at 1e-7. Input features were the 1-letter amino acid characters recoded as integers while the target values were the individual fold drug resistance ratios. After initial runs using all drug target values at once for training the regression model, large MSE values were obtained (not shown), which redirected analysis towards building individual trained matrices for each drug target. As a requirement for the MATLAB’s newff function, both the feature vectors and their matching target values were transposed. The number of hidden layers was varied from 1 to 3 while nodes were set at permutations of 2, 4, 6, 8 and 10 for each hidden layer. One hidden layer of 5–20 nodes was re-evaluated in cases where high training performances were observed to have a significantly lower test performances or high variances.

### Evaluation of training performance

Training performance was assessed both by regression and classification methods. For regression-based evaluation, the coefficient of determination (R^2^) values were obtained between the predicted (*y*_*i*_) and actual (*x*_*i*_) fold scores for the whole dataset using the formula

$$ {R}^2=\frac{{\left[n\left(\sum_{i=1}^n{x}_i{y}_i\right)-\left(\sum_{i=1}^n{x}_i\right)\left(\sum_{i=1}^n{y}_i\right)\right]}^2}{\left[n\ \sum_{i=1}^n{x}_i^2-{\left(\sum_{i=1}^n{x}_i\right)}^2\right]\left[n\ \sum_{i=1}^n{y}_i^2-{\left(\sum_{i=1}^n{y}_i\right)}^2\right]} $$

Further, the dataset was randomly divided into 5 subsets of approximately equal size, and 5 different ANNs were trained on datasets that comprised 4 of the 5 subsets, and then 5 different R^2^ values were calculated; we then calculated the mean and the standard deviation of these 5 R^2^ values. Regression performances were then compared against prediction models from the article published in 2016 by Shen and co-workers [19], in which regression machine learning models, namely the Random Forest and the K-nearest neighbor algorithms were used. The raw dataset used in this work and in ref. [19] is the same, i.e. the Stanford HIVdb dataset; however, the filtering used in this paper is as described above, whereas ref. [19] uses filtering provided by Stanford HIVdb [23]. In order to further verify our models against overfitting, R^2^values were calculated over different subsets of the data set, namely the whole dataset, the validation set and finally the test set.

Furthermore, classification accuracy was evaluated against Stanford HIVdb and a recently-published approach implemented as the SHIVA web server [17]. We used the EMBOSS backtranseq tool [25] to back-translate protein sequences to one of its (DNA) codon permutations in FASTA format as input for Stanford HIVdb’s Sierra web service (GraphQL API) tool to obtain resistance predictions. SHIVA predictions were obtained by submitting FASTA-formatted protein sequences to the web server. Drug resistance classes (susceptible, resistant and intermediate) were coded as numbers 0, 1 and 2 respectively. While Stanford HIVdb defined three classes, SHIVA defined two: susceptible and resistant. Classification accuracies were evaluated by calculating misclassification rates, defined as the proportion of non-concordant pairs between PhenoSense Assay classes and the independently-predicted classes for each of: our ANN approach, Stanford HIVdb and SHIVA. Cut-offs from Stanford HIVdb available at [26] were used for classifying our ANN predictions and those of the PhenoSense Assay dataset. We do not define new binary cut-offs for evaluating SHIVA; for a limited number of ARVs binary cut-offs are available from the PhenoSense Assay [27], and for the remaining ARVs we proceed in the following way. An upper and a lower bound misclassification rate were computed for SHIVA as the conversion from a multiclass to a binary classification is ambiguous - an intermediate class may lie closer to a resistant or susceptible class. We set the number of truly misclassified pairs (0,1 or 1,0) as the lower bound, while the number of discordant pairs involving intermediate resistance sequences (2,0 or 2,1) was added to the discordance value to set an upper bound for misclassification rates. All proportions were then evaluated as percentages, as shown in Table 2.

**Table 2:** Comparison of misclassification rates (percentages) for our ANN approach, Stanford HIVdb and SHIVA

ARVs		ANN	HIVdb	SHIVA
PIs	ATV	26.61	28.57	84.53
	DRV	2.98	22.57	32.41–53.49
	FPV	16.08	36.97	67.0–79.74
	IDV	34.29	26.19	81.92
	LPV	9.79	36.82	68.05–83.51
	NFV	25.23	20.36	80.84
	SQV	30.37	38.75	67.25–88.16
	TPV	9.07	39.88	unavailable
NRTIs	3TC	3.87	12.09	90.21
	ABC	6.53	33.78	50.76–72.25
	AZT	36.19	29.88	90.38
	D4T	7.31	44.07	79.15
	DDI	8.05	57.52	34.14–92.44
	TDF	5.39	37.2	37.36–66.53
NNRTIs	EFV	16.08	21.05	81.32
	ETR	6.58	13.21	unavailable
	NVP	24.87	9.4	73.97
	RPV	1.55	24.99	8.33

## Results and discussion

Table 1 shows that differing numbers of sequences were obtained from the different filtering approaches. In general, allowing expansion of sequences to less than 1000, combined with rare variant filtering produced the best results. Multiple (2–3) hidden layers were found to be required for all ARVs, with the exception of ABC, AZT, and RPV. DRV, ETR and RPV have the lowest numbers of unique sequence IDs, and hence may suffer from lack of generalizability compared to the other ARVs. However, in this study we attempted to find the optimal balance between the number of sequences and the possibility of retaining sequences containing sequencing errors.

The procedure used to build our models is referred to as protocol A. Our results are compared to the models used by Shen and co-workers [19], namely the Random Forest (RF) and the K-nearest neighbor (KNN), which both utilise Delaunay triangulation for structural feature encoding (henceforth referred to as protocol B and C respectively in this paper).

### Regression performances for HIV PIs

**Fig. 1:**
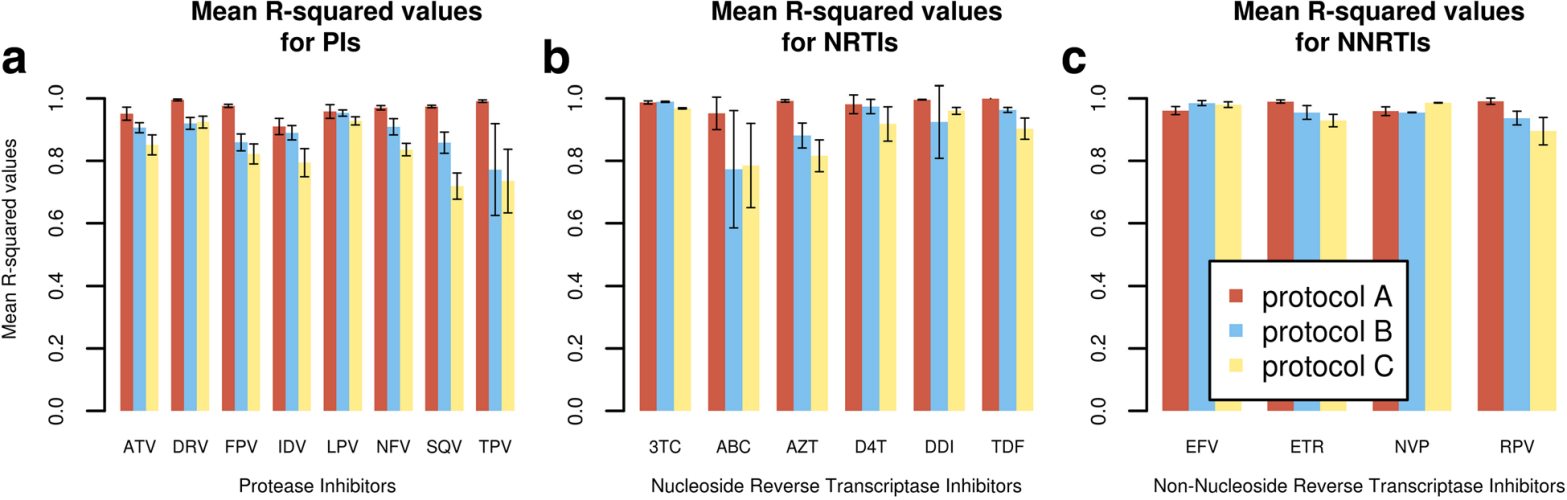
The mean R^2^ values and their standard deviations for the protocols A, B, C, and the various ARVs

The results are presented in Fig. 1a and Additional file 1: Table S1. The procedure used to build our models is referred to as protocol A. In all, protocol A yielded better results than protocols B and C. Very low variances were generally observed using protocol A, except in the case of ATV, IDV and LPV where variances were comparable to those observed in protocols B and C. Improvements of largest magnitudes for PIs were observed from protocol A for FPV, SQV and TPV with mean differences of 0.117, 0.116 and 0.219 respectively from the top-scoring protocols in B.

### Regression performances for NRTIs

In the case of NRTIs (Fig. 1b and Additional file 1: Table S2), better predictability was observed for all drugs using protocol A except for 3TC, where the performance, though high, was similar to that obtained in protocol B. Very high mean R^2^ values with very small variances were obtained for AZT, DDI and TDF. Their high degree of fit combined to their low variability suggests that the ANN model is explaining most of the observed variation, likely due to higher sequence quality obtained after filtering.

### Regression performances for NNRTIs

In the case of NNRTIs (Fig. 1c and Additional file 1: Table S3), protocol C outperformed protocol A by a narrow margin in for EFV and NVP. Very high mean accuracies were attained in the case of RPV and ETR, surpassing both protocols B and C. However, the smaller sample size for RPV (Table 1) (169 unique sequence IDs for a total of 2977 expanded sequences) may indicate that while appearing to perform exceptionally well, the model may not generalize well to more divergent sequences. ETR is supported by a comparatively higher number of unique sequence IDs, and will generalize slightly better that the model developed for RPV.

### Overfitting assessment

As seen in Table 3, for all ARVs we verify that overfitting is minimized by ensuring that R^2^ values do not significantly decline in the test set with respect to both the whole dataset and the validation sets.

**Table 3:** R^2^ values (3 dp) obtained from individual subsets obtained after filtering

ARV classes	ARVs	Whole dataset R^2^ values	Validation set R^2^ values	Test set R^2^ values
PIs	ATV	0.951	0.913	0.856
	DRV	0.991	0.991	0.989
	FPV	0.980	0.938	0.958
	IDV	0.899	0.816	0.842
	LPV	0.966	0.922	0.883
	NFV	0.975	0.924	0.939
	SQV	0.977	0.949	0.906
	TPV	0.989	0.995	0.943
NRTIs	3TC	0.995	0.988	0.985
	ABC	0.984	0.956	0.954
	AZT	0.994	0.979	0.985
	D4T	0.995	0.996	0.979
	DDI	0.997	0.997	0.992
	TDF	0.999	1.000	0.992
NNRTIs	EFV	0.976	0.905	0.967
	ETR	0.996	0.993	0.982
	NVP	0.962	0.939	0.927
	RPV	0.982	0.956	0.915

### Classification performance for all antiretrovirals

We provide additional support for our approach by comparing misclassification rates against Stanford HIVdb and SHIVA, all with respect to the PhenoSense assay data. It can be observed from Table 2 that lower misclassification rates are obtained, with the exception of NVP, AZT, NFV and IDV. An important point to observe here is that we considered the entirety of the dataset filtered by our means for the development of the ANN described in this paper, the counts being shown in Table 1. This was performed so that only high confidence sequences would be compared for each individual antiretroviral. Both Stanford HIVdb and SHIVA were developed using another data set, the Stanford HIVdb pre-filtered data, and this factor may have affected their performance on the dataset used here.

## Conclusions

This work focused on the pre-processing and optimization of ANN regression models for the prediction of fold resistance scores for HIV-1 subtype B using RT and PR PhenoSense data available in the public domain from Stanford HIVdb. As expressed by Dahake and co-workers [28], there is a need to develop subtype-specific databases, and we made such an attempt by constraining the dataset for subtype specificity, sacrificing generalizability for a higher predictive performance for subtype B. The results obtained show that the predictive quality of the ANN regression models is at least comparable to that of other methods, and for most ARVs is a definite improvement.

The approach presented in this paper is applicable to subtype B, and an obvious question is whether it can be extended to the other subtypes? Previous studies [29, 30] involving HIV-1 subtypes A, B and C envelope glycoprotein V3 loop region, suggest that subtype B and C share similar co-receptor usage as opposed to subtype A. Also, Raymond and co-workers [31]⁠ hinted that subtypes B and C share similar genotypic determinants, and for this reason, by extrapolation our method may extend to the C subtype. However, a key difficulty is the paucity of publicly available phenotypic assay data for training and testing any extrapolation to other subtypes, so the development of a methodology that leads to accurate models will be challenging [32, 33]. It is hoped that our work will lead to more non-B subtype drug resistance data becoming available.

## Additional file

Additional file 1:
**Table S1.** Mean R2 values and their standard deviations for PIs for protocols A, B and C. **Table S2.** Mean R2 values and their standard deviations for NRTIs for protocols A, B and C. **Table S3.** Mean R2 values and their standard deviations for NNRTIs for protocols A, B and C. (DOC 45 kb)

## Abbreviations

3TC: Lamivudine
ABC: Abacavir
ANN: Artificial neural network
ANRS: Agence Nationale de Recherche sur le Sida et les hepatites virales
ARV: Antiretroviral
ATV: Atazanavir
AZT: Zidovudine
D4T: Stavudine
DDI: Didanosine
DRV: Darunavir
EFV: Efavirenz
ETR: Etravirin
FPV: Fosamprenavir
HIV: Human immunodeficiency virus
HIVdb: HIV drug resistance database
IDV: Indinavir
KNN: K-Nearest Neighbors
LPV: Lopinavir
MSE: Mean squared error
NFV: Nelfinavir
NNRTI: Non-nucleoside reverse transcriptase inhibitor
NRTI: Nucleoside reverse transcriptase inhibitor
NVP: Nevirapine
PI: Protease inhibitor
RF: Random forest
RPV: Rilpivirine
RT: Reverse transcriptase
SQV: Saquinavir
TDF: Tenofovir
TPV: Tipranavir
