# Supplementary material for: Improving fold resistance prediction of HIV-1 against protease and reverse transcriptase inhibitors using artificial neural networks
Source: BMC Bioinformatics. 2017 Aug 15;18:369. doi: 10.1186/s12859-017-1782-x (PMC5558779; doi:10.1186/s12859-017-1782-x)
Supplement: Additional file 1: — Table S1. Mean R2 values and their standard deviations for PIs for protocols A, B and C. Table S2. Mean R2 values and their standard deviations for NRTIs for protocols A, B and C. Table S3. Mean R2 values and their standard deviations for NNRTIs for protocols A, B and C. (DOC 45 kb) [file 12859_2017_1782_MOESM1_ESM.doc]

## Supplementary Tables

| **Table S1: Mean R^2^ values and their standard deviations for PIs for protocols A, B and C.** | | | |
| --- | --- | --- | --- |
| **ARVs** | **Protocol A** | **Protocol B** | **Protocol C** |
| **ATV** | 0.951 ± 0.021 | 0.906 ± 0.016 | 0.851 ± 0.032 |
| **DRV** | 0.995 ± 0.003 | 0.92 ± 0.019 | 0.924 ± 0.019 |
| **FPV** | 0.976 ± 0.005 | 0.859 ± 0.027 | 0.822 ± 0.032 |
| **IDV** | 0.910 ± 0.026 | 0.89 ± 0.023 | 0.794 ± 0.045 |
| **LPV** | 0.958 ± 0.022 | 0.953 ± 0.01 | 0.928 ± 0.013 |
| **NFV** | 0.970 ± 0.007 | 0.909 ± 0.026 | 0.836 ± 0.02 |
| **SQV** | 0.974 ± 0.004 | 0.858 ± 0.034 | 0.719 ± 0.042 |
| **TPV** | 0.991 ± 0.004 | 0.772 ± 0.147 | 0.735 ± 0.102 |

| **Table S2: Mean R^2^ values and their standard deviations for NRTIs for protocols A, B and C.** | | | |
| --- | --- | --- | --- |
| **ARVs** | **Protocol A** | **Protocol B** | **Protocol C** |
| **3TC** | 0.987 ± 0.005 | 0.989 ± 0.002 | 0.968 ± 0.002 |
| **ABC** | 0.952 ± 0.052 | 0.773 ± 0.188 | 0.785 ± 0.135 |
| **AZT** | 0.992 ± 0.004 | 0.881 ± 0.04 | 0.816 ± 0.051 |
| **D4T** | 0.981 ± 0.030 | 0.974 ± 0.023 | 0.918 ± 0.055 |
| **DDI** | 0.996 ± 0.001 | 0.924 ± 0.116 | 0.96 ± 0.011 |
| **TDF** | 0.9996 ± 0.0002 | 0.963 ± 0.008 | 0.903 ± 0.034 |

| **Table S3: Mean R^2^ values and their standard deviations for NNRTIs for protocols A, B and C.** | | | |
| --- | --- | --- | --- |
| **ARVs** | **Protocol A** | **Protocol B** | **Protocol C** |
| **EFV** | 0.961 ± 0.013 | 0.985 ± 0.008 | 0.980 ± 0.009 |
| **ETR** | 0.990 ± 0.005 | 0.955 ± 0.022 | 0.929 ± 0.02 |
| **NVP** | 0.959 ± 0.014 | 0.955 ± 0.001 | 0.986 ± 0.001 |
| **RPV** | 0.991 ± 0.010 | 0.937 ± 0.022 | 0.895 ± 0.044 |
